# A particle-filled hydrogel based on alginate and calcium phosphate nanoparticles as bone adhesive

**DOI:** 10.1007/s10856-024-06798-8

**Published:** 2024-10-14

**Authors:** Benedikt Kruse, Katarina Vasic, Kai O. Böker, Arndt F. Schilling, Wolfgang Lehmann, Matthias Epple

**Affiliations:** 1 Inorganic Chemistry and Centre for Nanointegration Duisburg-Essen (CENIDE), University of Duisburg-Essen, Essen, Germany; 2 Clinic for Trauma Surgery, Orthopedics and Plastic Surgery, University Medical Center Goettingen, Goettingen, Germany

## Abstract

The clinical need for bone adhesives as an alternative to osteosynthesis is evident. However, this is a challenging problem due to the moist environment in surgical sites with bone surfaces covered with blood and biomolecules like lipids or proteins. A nanoparticle-loaded hydrogel that is based on a freeze-dried powder of silica-coated calcium phosphate/carboxymethyl cellulose nanoparticles (CaP/CMC/SiO_2_) and an aqueous solution of sodium alginate (2 wt%) was developed and optimized with respect to the gluing ability in air and in water. The final paste was crosslinked within about one minute by calcium ions released from the calcium phosphate nanoparticles and contained about 20 wt% nanoparticles and 80 wt% water. The mechanical properties of the hydrogel were determined by extensive rheological tests. The thixotropic pasty hydrogel can be applied with a syringe. The adhesion strength was about 84 kPa between moist bone fragments in air. The hydrogel kept fragments of cortical bone well connected for >3 months during complete submersion in water. Besides water, the material consists only of biocompatible and biodegradable components (calcium phosphate, CMC, alginate). It carries only a very low dose of these materials into the bone site (mainly calcium phosphate nanoparticles). In-vitro cell culture with hMSCs that differentiated to osteoblasts confirmed a good biocompatibility of the bone adhesive formulation.

**Graphical Abstract:**

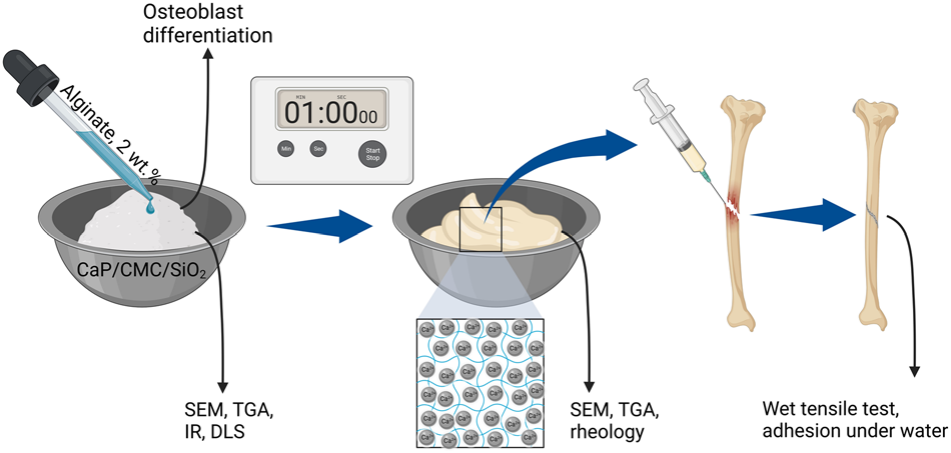

## Introduction

Bone fractures are usually treated by osteosynthesis, i.e., by screws, plates, or nails. A fixation by a suitable bone adhesive would allow a direct fusion without the need for a metallic or polymeric implant. It would also permit to join small bone fragments where osteosynthesis is geometrically difficult, e.g., in a complicated fracture site. However, the task to glue bone in vivo is very challenging because bone has a wet surface and the presence of blood, biomolecules (proteins, lipids etc.), and cells compromises an efficient adhesion. Besides the mechanical requirements for the adhesion itself, aspects like biocompatibility and biodegradability also must be taken into account. Ideally, a bone adhesive should not persist at the interface but permit the ingrowth of new bone to join the bone fragments ad integrum with a minimum amount of foreign material. The general requirements for a bone adhesive have been outlined in a number of review articles [1–4].

A wide range of bone adhesives has been proposed, e.g., inorganic cements consisting of calcium phosphate [5], magnesium phosphate [6], or glass ionomer cements [7]. Organic glues are mainly based on polymers like polyurethanes [8, 9], polycyanoacrylates [10], polymethyl methacrylates [11, 12], and poly(2-oxazoline)s [13] that can be chemically or photochemically crosslinked. Modifications of polymers with bio-inspired animal-derived bone-adhesive groups, e.g., peptides and dihydroxyphenylalanine (DOPA) from mussels [14] and sandcastle worms [15] are also of interest. Polymeric adhesives from natural sources are usually based on fibrin [16] or polysaccharides like chitosan [17], alginate [18], and gelatine [1, 19]. Commercial cements for bone fixation based on a mixture of the non-physiological amino acid phosphoserine and the calcium phosphate ceramic α-tricalcium phosphate (α-TCP) [20] or tetracalcium phosphate [21, 22] have been developed. Modifications have combined phosphoserine with magnesium phosphates [23]. A strong gluing effect was reported for a combination of poly(vinyl alcohol), the amino acid L-DOPA, and a metal-organic framework (MOF), i.e., zeolitic imidazolate framework-8 (ZIF-8) [24].

Up to date, none of the above systems has fulfilled all the above-mentioned clinical requirements. Therefore, we have focused on the creation of a biocompatible hydrogel based on alginate and calcium phosphate nanoparticles that also allows local bone formation in the former defect. A mixture of calcium phosphate nanoparticles [25] with an aqueous solution of alginate [18] gives a hydrogel where the calcium ions that are released from the calcium phosphate nanoparticles lead to cross-linking of the alginate. Alginate thus acts as glue to combine two bone fragments. Our concept relies exclusively on biodegradable and biocompatible components with the beneficial effect of calcium phosphate nanoparticles (inorganic part of bone) in bone contact. The material does not start to harden immediately after mixing like a bone cement but remains applicable as viscous paste without time restriction, e.g., with a syringe. The low solid-to-liquid ratio corresponds to a low synthetic amount of biomaterial in the bone defect, with most of it bone mineral-like calcium phosphate nanoparticles.

## Materials and methods

### Chemicals

Calcium lactate pentahydrate (>98%), alginic acid sodium salt (Alg, *MW* 300–350 kDa), and carboxymethylcellulose, sodium salt (CMC, *MW* 90 kDa) were obtained from Carl Roth (Karlsruhe, Germany). Diammonium hydrogen phosphate ((NH_4_)_2_HPO_4_, >99%), tetraethyl orthosilicate (TEOS, >99%), ammonia solution (28 wt% NH_3_ in water), and sodium hydroxide (NaOH, >98%) were obtained from Sigma-Aldrich (St. Louis, MO, USA). Ethanol (EtOH, 99.8%) was obtained from ThermoFisher Scientific (Darmstadt, Germany). Ultrapure water (Purelab ultra instrument, ELGA) with a specific resistivity of 18.2 MΩ was used for all syntheses and glueing experiments unless otherwise noted.

### Synthesis of the nanoparticle-loaded alginate hydrogel

The hydrogel was formed by mixing calcium phosphate nanoparticles with an aqueous alginate solution. For the synthesis of CMC-coated calcium phosphate nanoparticles (CaP/CMC), aqueous solutions of calcium lactate (100 mL, 62.5 mM) and diammonium hydrogen phosphate (100 mL, 37.4 mM) were adjusted to pH 10 with NaOH (0.1 M). Furthermore, an aqueous solution of carboxymethylcellulose (CMC, 22 mL, 2 g L^−1^) was prepared. These three solutions were mixed rapidly in a round bottom flask under stirring. After 20 min stirring, the formed CaP/CMC nanoparticles were coated without prior isolation with a silica shell in a modified Stoeber process as reported earlier [26]. For this, a solution of 8 mL TEOS in 888 mL ethanol and 4 mL of an NH_3_ solution (7.8 wt%) were quickly added. The reaction mixture was stirred for 16 h at ambient temperature. The silica-coated nanoparticles (CaP/CMC/SiO_2_) were isolated from the dispersion by centrifugation for 15 min at 4000 rpm, followed by washing with water. The material was freeze-dried in a Christ Alpha 2–4 LSC device (Martin Christ GmbH, Osterode am Harz, Germany) to remove adherent water. The white CaP/CMC/SiO_2_ powder was gently ground and stored at 4 °C in a closed container under exclusion of moisture.

The nanoparticles were redispersed in water and characterized by dynamic light scattering (DLS) combined with zeta potential measurements to determine the hydrodynamic diameter and dispersion stability (Zetasizer Nano ZS, Malvern Panalytical, Kassel, Germany). Scanning electron microscopy (SEM) was used to obtain the particle size and morphology (ESEM Quanta 400 FEG microscope, ThermoFisher, Hillsboro, OR, USA). All SEM samples were sputtered for 20 s with a gold-palladium alloy before measurement. Atomic absorption spectroscopy (AAS; iCE 3000 M-Series spectrometer, Thermo Scientific, Waltham, MA, USA) was performed to determine the calcium content of the nanoparticles after dissolution in aqueous HCl. Thermogravimetry (STA 449 F3 Jupiter, Netzsch, Selb, Germany) was performed at a heating rate of 5 K min^−1^ from 25 to 900 °C in an alumina crucible under dynamic oxygen atmosphere (50 mL min^−1^). Infrared spectroscopy (IR) was performed with an ATR Alpha Platinum spectrometer (Bruker, Billerica, MA, USA).

The alginate/CaP hydrogel was obtained after mixing the freeze-dried CaP/CMC/SiO_2_ powder with alginate solutions with different concentrations of sodium alginate (0.5, 1.0 and 2.0 wt%) until a pasty consistency was reached. This happened within about one minute, i.e., the gelation was rapidly finished. After that, the mechanical properties of the paste did not change with time provided no drying occurred. For application as glue, the paste was transferred with a spatula into a syringe for injection to the area of interest. Rheological studies of the paste were conducted with a modular compact rheometer (MCR301, Anton Paar, Ostfildern, Germany). For tensile testing of adhesion, a material testing machine (EZ SX, Shimadzu, Kyoto, Japan) was used.

CaP/CMC/SiO_2_ circular discs were prepared to assess the cytocompatibility of the bone adhesive. A total of 20 mg dry powder was compressed in a KBr pressing mould (Maaßen, Reutlingen, Germany) with 2 t with a hydraulic laboratory press (Perkin-Elmer, Waltham, MA, USA) to discs of 10 mm diameter and 0.3 mm thickness.

Cortical bovine bone was used as model surface to test the adhesion of different paste compositions. The thoroughly rinsed tubular bones were cut with a circular saw into discs with a height of about 2 cm and subsequently quartered into smaller fragments. For tensile tests, planar bone slices were prepared by cutting the tubular bone lengthways into equal parts with a circular saw, followed by a fine-tuning of the size with a low-speed saw. After the paste was applied to the two bone fragments, both were pressed together with a force of 3.67 N for 30 s and wetted with water to simulate biological conditions. Subsequently, the tensile test was conducted at a speed of 1 mm min^−1^.

### Cell culture studies

A single cell-derived human mesenchymal stem cell line (Single clone pick 1 (SCP1) [27]) expressing the human telomerase reverse transcriptase (hTERT) was originally obtained from Cambrex (East Rutherford, NJ, USA). The cells were cultivated in 25 cm^2^ cell culture flasks in low-glucose DMEM (Gibco, Waltham, MA, USA) supplemented with 10% fetal bovine serum (FBS) and 1% penicillin/streptomycin. Prior to the induction of the osteogenic differentiation, the cells were seeded in 24-well plates with 10^4^ cells cm^−2^ seeding density either on the surface of CaP/CMC/SiO_2_ circular discs or directly in the well as control and allowed to attach overnight. The medium was then changed to osteoblast differentiation media, i.e., low-glucose DMEM supplemented with 10% FBS, 1% penicillin/streptomycin, 200 µM ascorbic acid-2 phosphate, 10 mM β-glycerophosphate, and 0.1 µM dexamethasone. The medium was changed three times per week for 28 days. Then, the cells were fixed and stained with Sirius Red staining according to the manufacturer’s protocol (Chondrex Inc., Woodinville, WA, USA).

Images of the wells were obtained with a Leica DMi 8 microscope at 10× magnification for each biological replicate (*n* = 6 for each group). Pixel classification by Ilastik [28] was used to distinguish stained from unstained areas which were then quantified by ImageJ [29]. The numerical results are given as mean values of the percentage of the stained area together with the standard deviation.

## Results

Freeze-dried CMC-stabilized and silica-coated calcium phosphate nanoparticles, CaP/CMC/SiO_2_, were mixed with a sodium alginate solution in obtain an injectable pasty hydrogel as bone adhesive. The biopolymer carboxymethylcellulose was used to stabilize the calcium phosphate nanoparticles to prevent crystal growth and particle aggregation. CMC is non-toxic, approved for food applications [30] by the Federal Drug Administration (FDA, USA) and therefore be a suitable reagent for biomedical application. Calcium phosphate constitutes the inorganic part of human bone and is highly biocompatible, osteoconductive, and biodegradable [25, 31–34]. The silica shell was added in a modified Stoeber process to increase the integrity of the particles and to avoid disintegration in an aqueous environment. The release of calcium ions by the calcium phosphate nanoparticles induced gelation of the alginate due to the incorporation of calcium cations into the mannuronic acid part of the sodium alginate [35]. Notably, control experiments with CaP/CMC particles without a silica shell together with alginate generally resulted in poor adhesion properties, therefore this direction was not further explored.

**Fig. 1:**
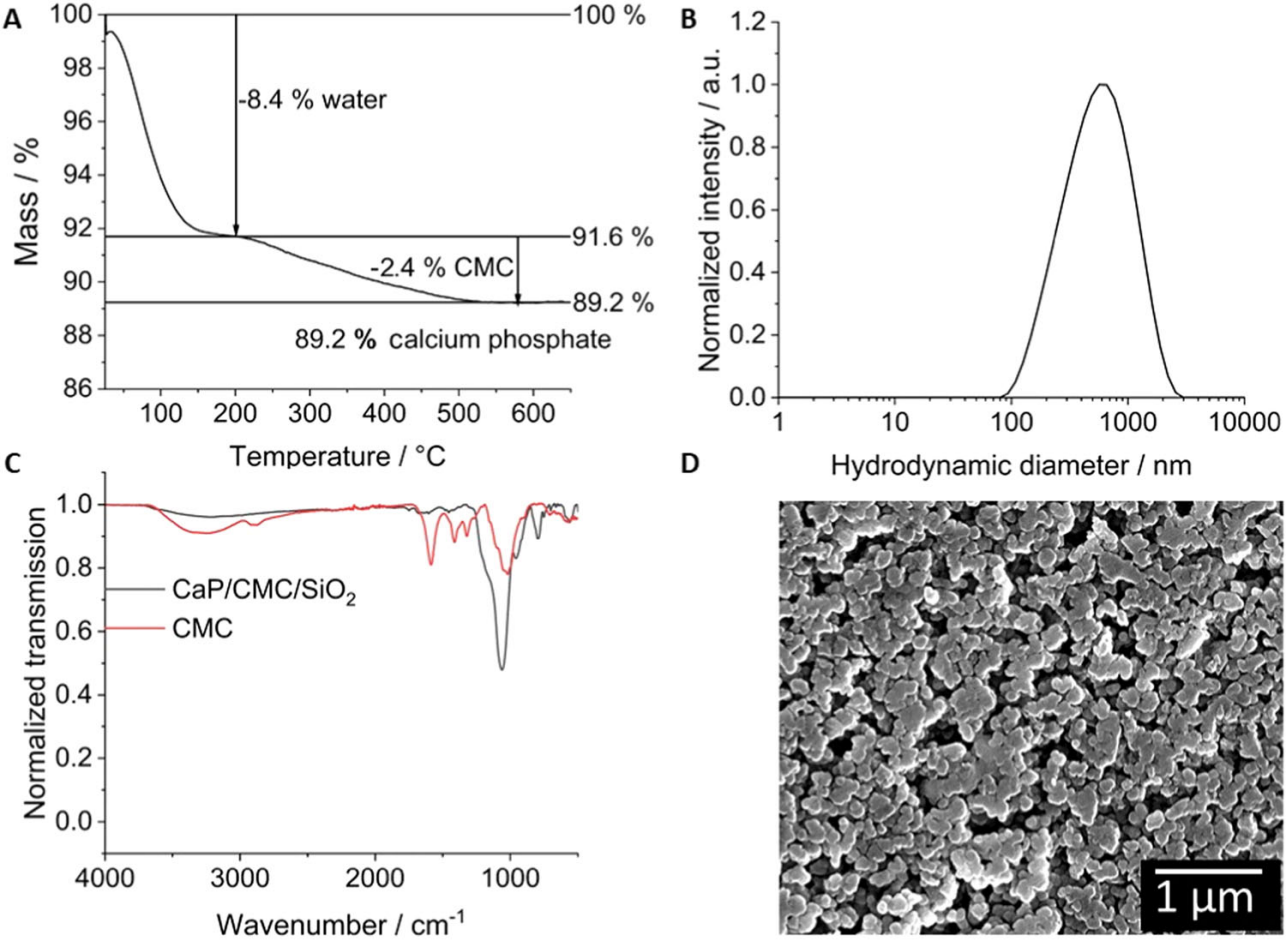
Chemical characterization of the freeze-dried CaP/CMC/SiO_2_ nanoparticles. **A** Thermogravimetric analysis. **B** Dynamic light scattering. **C** Infrared spectroscopy. **D** Scanning electron microscopy

The particles were investigated by dynamic light scattering (DLS), scanning electron microscopy (SEM), and thermogravimetry (TG) (Fig. 1). SEM images showed an approximately spherical morphology and an average particle size of 76 nm. Thermogravimetry showed that the freeze-dried particles consisted of 8.4 wt% residual water, 2.4 wt% CMC, and 89.2 wt% calcium phosphate/silica. The calcium content of the particles by AAS was 29 wt%. IR-spectroscopy confirmed the presence of CMC on the nanoparticles with characteristic bands. The broad band around 3436 cm^−1^ corresponds to O-H stretching vibrations (water), the band at 2922 cm^−1^ corresponds to the C-H stretching vibration (CMC), and the asymmetric band at 1609 cm^−1^ corresponds to the C = O stretching vibration (CMC). The bands at 1416 and 1332 cm^−1^ correspond to symmetric vibrations of alkyl groups (CMC), and the band at 1068 cm^−1^ corresponds to C-O-C stretching vibrations (CMC). Dynamic light scattering showed a negative particle charge due to the anionic polyelectrolyte CMC (zeta potential −24 mV) and a considerable degree of particle agglomeration as shown by the large particle size of 747 nm (hydrodynamic diameter).

The well-established polyelectrolyte alginate [18] formed the basis of the injectable bone adhesive. Mixing sodium alginate solution with the freeze-dried powder in defined proportion led to a viscous pasty hydrogel that was well applicable by a syringe. By adjusting the ratio of sodium alginate solution and powder (liquid-to-powder ratio, L/P, Table 1), the viscosity of the paste was controllable. Gelation occurred within about one minute after mixing in all cases, indicating a rapid release of calcium ions from the calcium phosphate nanoparticles to connect the alginate to induce gelation. Remarkably, no additional reagent like the acidifying agent glucono-δ-lactone (GDL) was required to induce a satisfactory gelation and interconnection [36].

**Table 1:** Nominal composition of the bone adhesive paste prepared with different sodium alginate concentrations, optimized for good viscous properties

	0.5 wt% alginate	1.0 wt% alginate	2.0 wt% alginate
CaP/CMC/SiO_2_ in the paste / wt%	14.3	16.4	19.9
CaP/SiO_2_ in the paste / wt%	13.9	16.0	19.4
CMC in the paste / wt%	0.4	0.4	0.5
sodium alginate in the paste / wt%	0.4	0.8	1.6
water in the paste / wt%	85.3	82.8	78.5
Mixing ratio of alginate solution to freeze-dried CaP/CMC/SiO_2_ nanoparticles (*m*:*m*)	5.4 : 1	4.6 : 1	3.6 : 1
Liquid-to-powder (L/P) ratio in the paste (*m*:*m*)	6.0 : 1	5.1 : 1	4.0 : 1

**Fig. 2:**
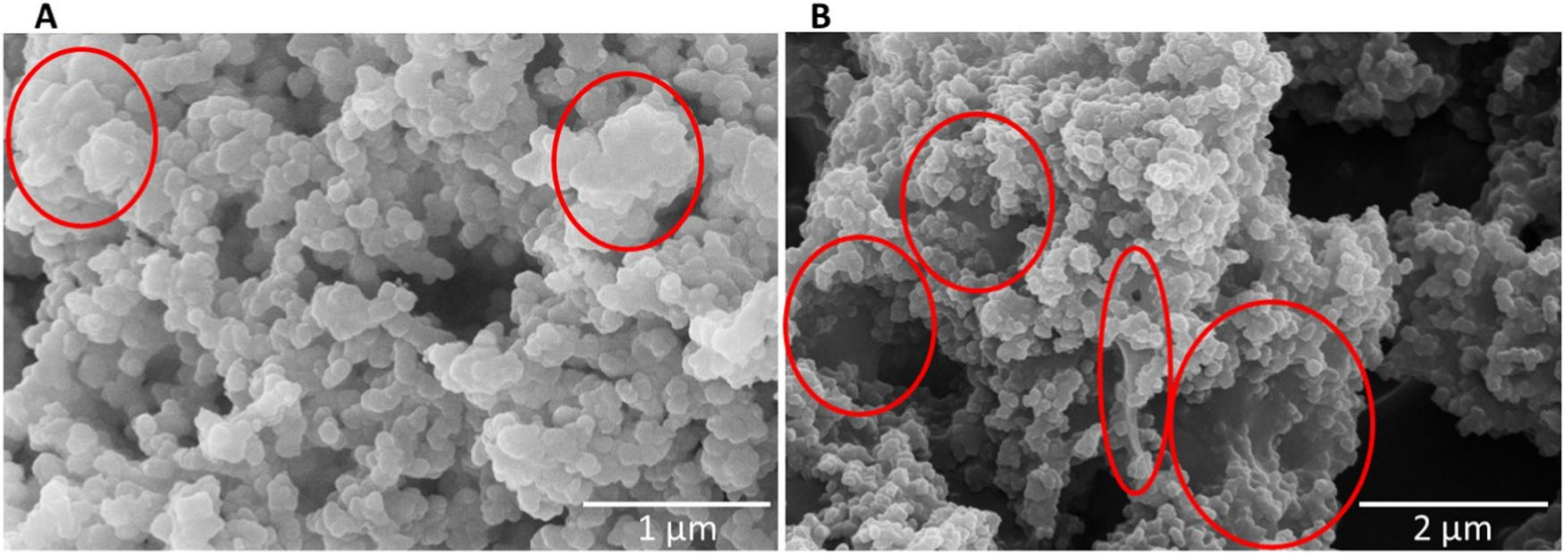
SEM images of the dried nanoparticle-loaded hydrogel prepared with 1 wt% **A** and 2 wt% **B** sodium alginate solution. Red circles indicate the presence of areas with high local alginate concentration, forming a polymer film connecting the particles

The calcium phosphate particles did not change after immersion in alginate as SEM clearly demonstrated. The previously dissolved alginate was visible as a thin layer on the particle surface after drying (Fig. 2).

We carried out extensive rheological measurements of the formed hydrogel immediately after mixing the nanoparticles with the alginate solution. Three pastes prepared with different L/P ratios (see Table 1) were analysed. All measurements were carried out in plate geometry with a 15 mm diameter plate at 37 °C. The paste was spread evenly onto the surface of the lower plate. The upper plate was lowered to a 1 mm measurement gap with the paste fully covering both plates. The gel structure was probed by an amplitude sweep experiment at a frequency of 10 rad s^−1^ and a deformation from 0.001 to 100% (100% corresponding to a 90° rotation).

**Fig. 3:**
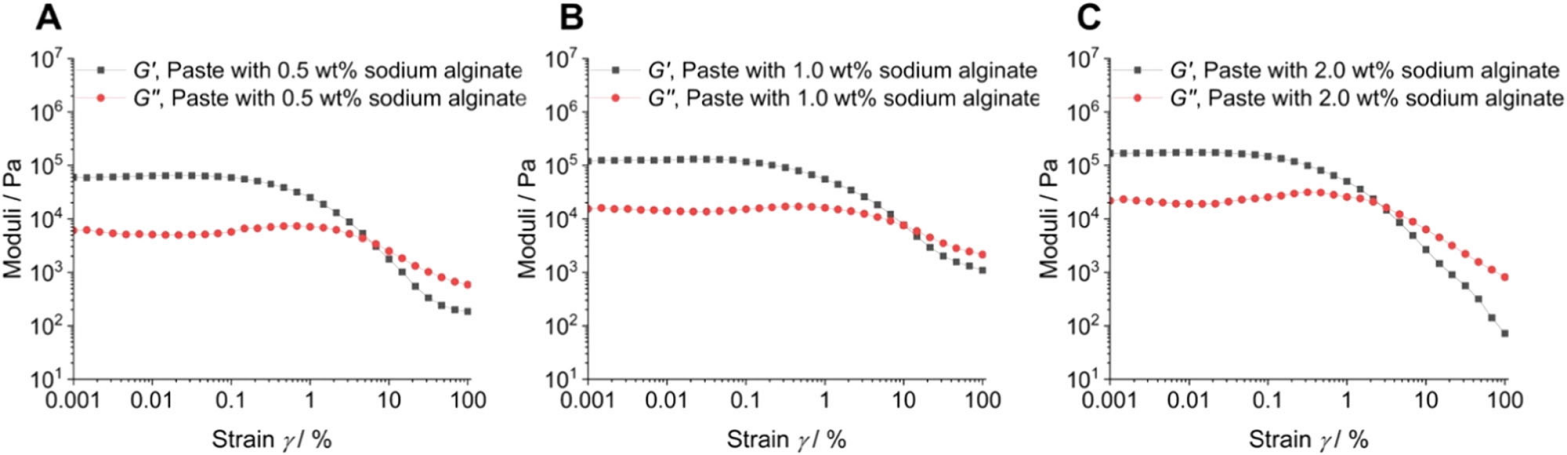
Amplitude sweep of different hydrogel compositions in plate geometry at 37 °C with a measurement gap of 1 mm. The three paste compositions are shown in panels **A**, **B**, and **C** as indicated.

All investigated samples showed a higher elastic modulus *G*’ in comparison to the viscous modulus *G*” (Fig. 3). Furthermore, the linear viscoelastic region (LVE) for each paste was derived from the amplitude sweep where *G*’ was independent of the applied deformation up to a strain of *γ* = 0.05%. The *G*’ values of the pastes increased with increasing sodium alginate content from 62 to 125 and 171 kPa, respectively. At strains of *γ* ≈ 1% the values for the loss modulus *G*” gave a shallow maximum for all paste compositions. This is a typical behaviour of highly concentrated dispersions, gels and crosslinked materials, indicating the breakup of internal crosslinks [37].

**Fig. 4:**
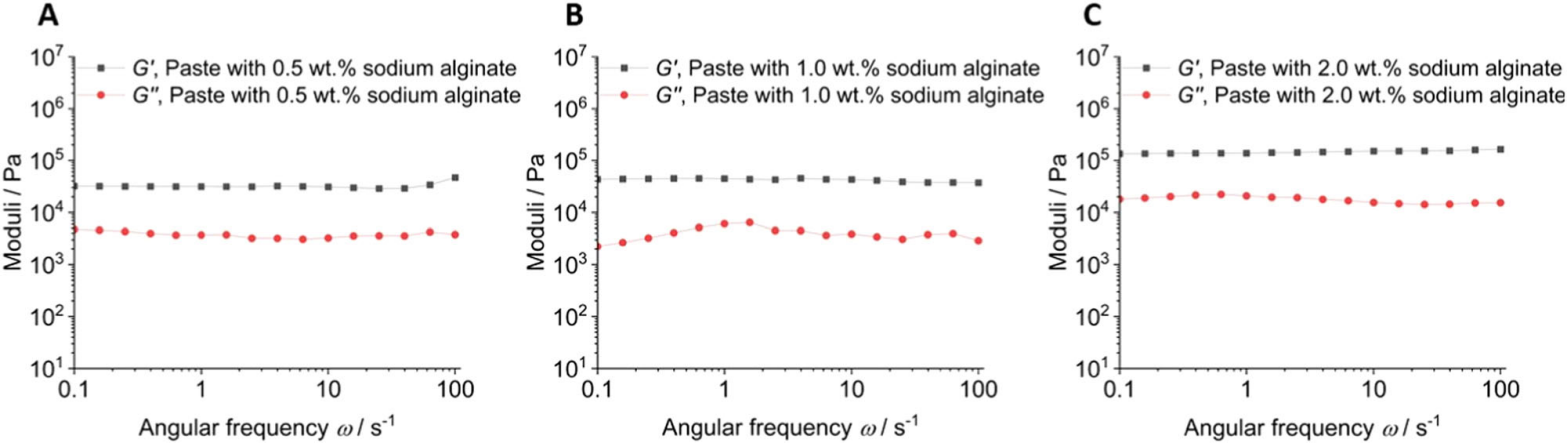
Frequency sweep experiment of different hydrogel compositions in plate geometry at 37 °C with a constant strain of *γ* = 0.05%. The three paste compositions are shown in panels **A**, **B**, and **C** as indicated.

Next, a frequency sweep experiment was carried out in controlled deformation mode within the linear viscoelastic (LVE) region of *γ*_LVE_ = 0.05% at angular frequencies from 0.1 to 100 s^−1^ (Fig. 4). In all experiments, the storage modulus *G*’ was always higher than the loss modulus *G*”, i.e., the elastic characteristics of the composite were more dominant than the viscous behaviour as it is typical for a gel. Over the whole frequency range, *G*’ and *G*” were almost independent on the frequency which indicates a high degree of crosslinking [35].

**Fig. 5:**
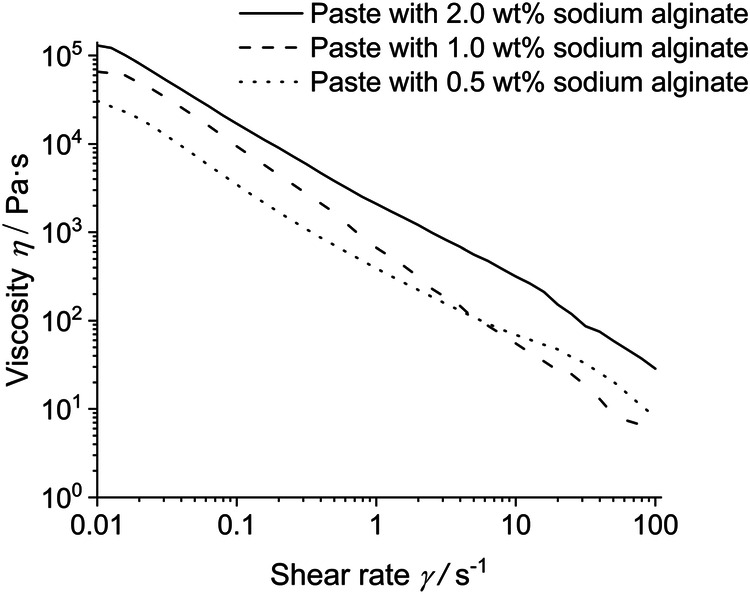
Flow curves of the hydrogels at constant strain of *γ* = 0.05% at 37 °C with increasing shear rate

Flow curves at variable shear rates are shown in Fig. 5. In all samples, a linear decrease in viscosity was observed with increasing shear rate.

**Fig. 6:**
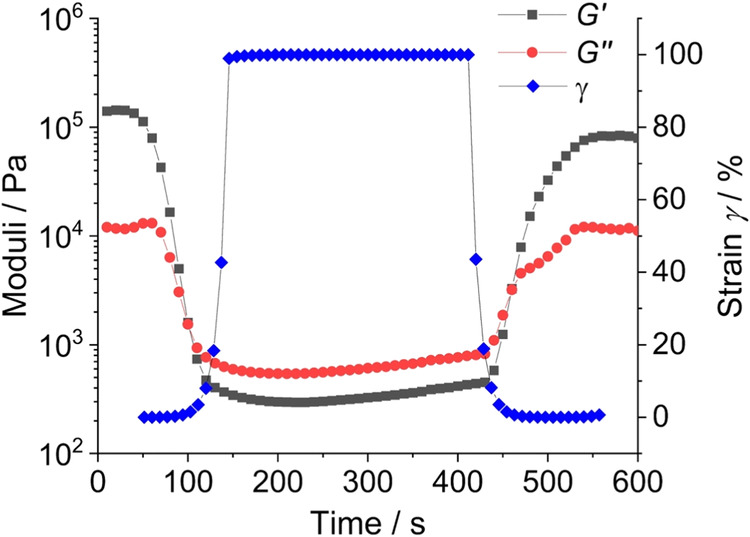
Thixotropy test of the nanoparticle-loaded hydrogel based on 2 wt% alginate in three amplitude sweep intervals (*ω* = 10 s^−1^). First section up to 100 s: Reference interval. Second section from 100 to 400 s: An increasing amplitude up to *γ* = 100% leads to structural disintegration. Third section after 400 s: Gel recovery phase

The paste with 2 wt% sodium alginate content showed the highest viscosity and therefore the highest degree of cross-linking. Thus, it was selected for all subsequent tests. A thixotropy test was carried out with this formulation to investigate the time-dependent recovery behaviour of the paste structure after mechanical loading during injection. A step test with three different amplitude sweeps was performed immediately after hydrogel preparation (Fig. 6). The first interval comprised an increasing amplitude from 0.01 to 100% at an angular frequency of *ω* = 10 s^−1^ for 120 s. In the second interval, the amplitude was kept constant at 100% for 300 s to break up the internal structure at *ω* = 10 s^−1^. During the third interval, the amplitude was decreased again from 100 to 0.01% within 180 s at *ω* = 10 s^−1^.

The paste showed mostly elastic properties with a storage modulus of *G*’ = 1.43·10^5 ^Pa in comparison to the loss modulus of *G*” = 1.17 × 10^4 ^Pa within the LVE range of *γ* = 0.05% at the start of the measurement. An increasing amplitude led to a loss of elastic properties and a complete breakdown of the gel network at *γ* = 100%. The viscous properties with *G*″ = 542 Pa were dominant over the elastic properties with *G*’ = 296 Pa. A reduction of the amplitude back to zero allowed the hydrogel network to rebuild. The thixotropy time to reach *G*′ = *G*″, i.e., for network restoration, was about 40 s. After 3 min, 60% (*G*’ = 8.29 × 10^4 ^Pa) of the initial gel stability was recovered.

**Fig. 7:**
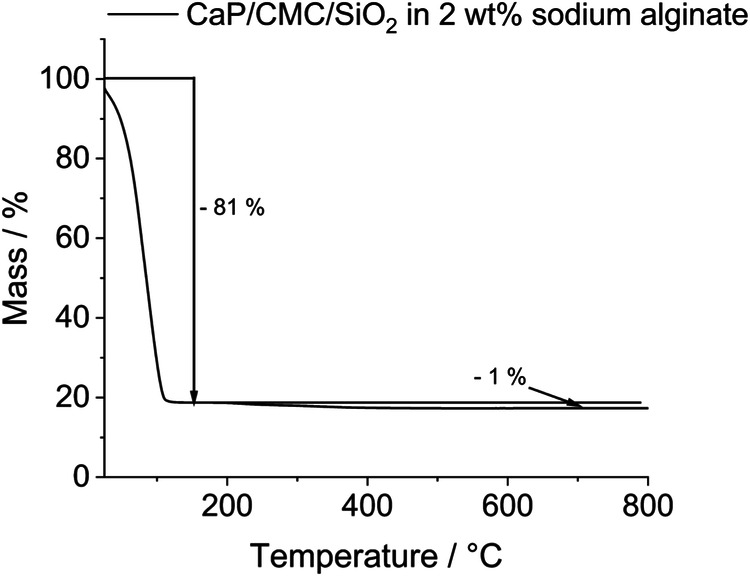
Thermogravimetric analysis of the nanoparticle-loaded hydrogel based on 2 wt% sodium alginate

**Fig. 8:**
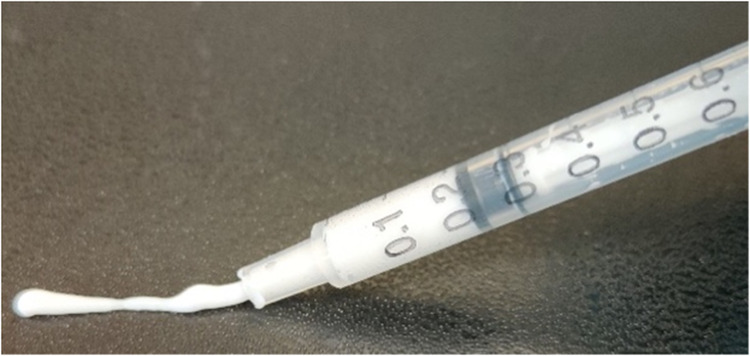
Demonstration of the application of the bone adhesive paste with a syringe. The hydrogel is thixotropic and therefore well applicable under mechanical stress

Thermogravimetry of the hydrogel based on 2 wt% sodium alginate gave a water content of 81 wt% (Fig. 7). The combustion of organic matter (alginate and CMC) led to a weight loss of about 1 wt%. The remaining 18 wt% correspond to the incombustible calcium phosphate/silica content in the hydrogel. The pasty hydrogel was well applicable with a syringe (Fig. 8).

**Fig. 9:**
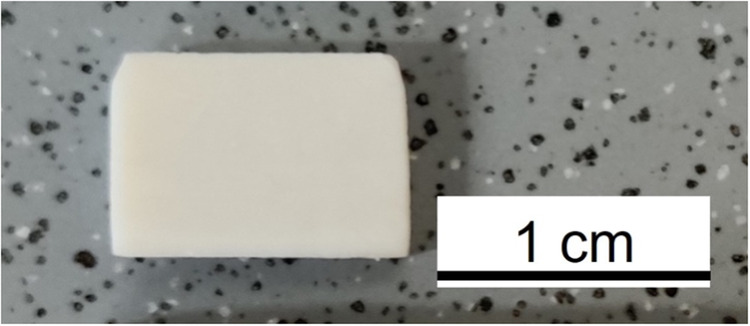
A cut piece of cortical bovine bone sample for adhesive testing

Testing bone adhesion strength and other aspects of the adhesive performance is complex and has not yet been standardized [20]. A wide range of both testing methods and clinical scenarios make it difficult to define minimum essential levels of adhesion [2]. For tensile tests, we prepared defined pieces of cortical bovine bone (Fig. 9) to obtain samples with plane-parallel surfaces.

**Fig. 10:**
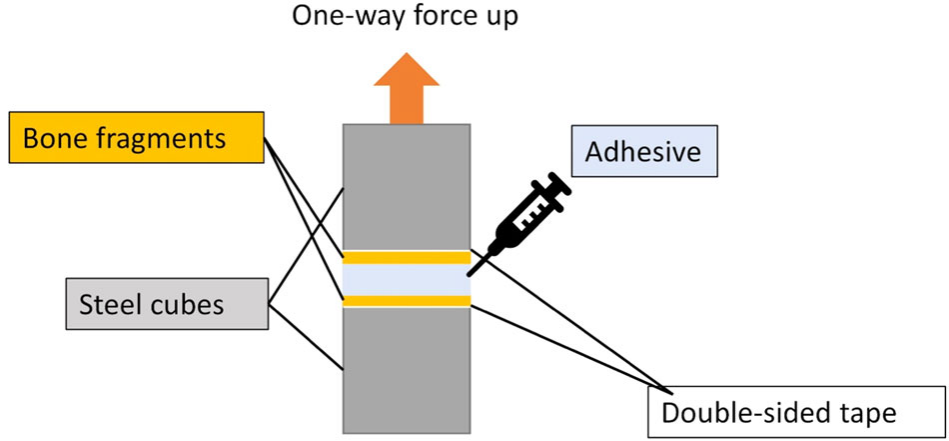
Experimental setup for tensile testing of the adhesive gel on plane-parallel bone fragments. The bone fragments were kept moist during the whole experiment by spraying with water

In our custom-made tensile testing setup (Fig. 10), two planar bone parts were aligned parallel and attached to steel cubes with double-sided adhesive tape. The paste was injected into the gap between the lower and the upper bone fragment. The bone fragments were immersed in water before the gluing and also kept moist from the outside throughout the experiment by spraying with water. The contact area was 0.55 cm^2^, and the thickness of the applied paste layer was about 0.2 mm.

**Fig. 11:**
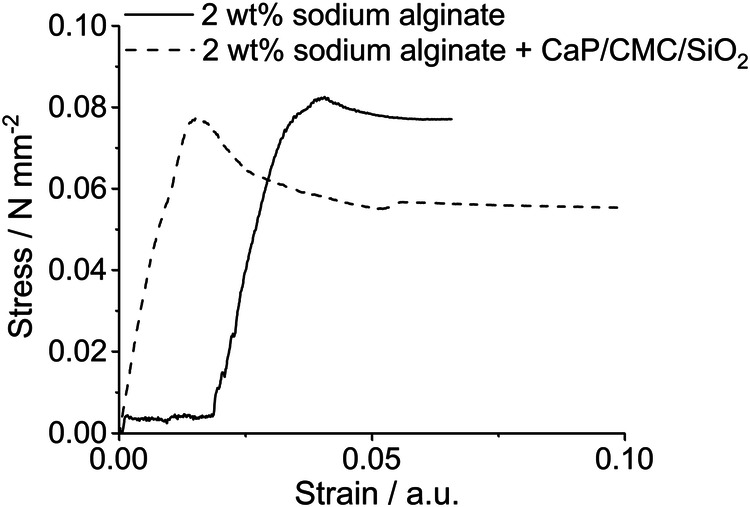
Stress-strain test of planar cut bone fragments glued with a hydrogel based on 2 wt% sodium alginate solution with and without calcium phosphate nanoparticle. The shift in the onset of the stress formation is due to the variations in the experimental setup shown in Fig. 10 where the distance of the glued bone plates was slightly different

2 wt% sodium alginate solution was used as glue with and without nanoparticles. A yield strength of 84 kPa was obtained in both cases (Fig. 11 and Table 2). This can be probably ascribed to comparable surface tension effects in both systems.

**Table 2:** Tensile testing results of bone glued with a hydrogel based on 2 wt% sodium alginate solution with and without calcium phosphate nanoparticles (gluing of moist planar bone fragments; contact area 0.55 cm^2^; *N* = 3)

Sample composition	Yield strength / kPa	Young’s modulus / MPa
2 wt% sodium alginate	84 ± 2	3.5 ± 1.8
2 wt% sodium alginate+calcium phosphate	83 ± 1	4.4 ± 0.2

The experimental setup is shown in Fig. 10

**Fig. 12:**
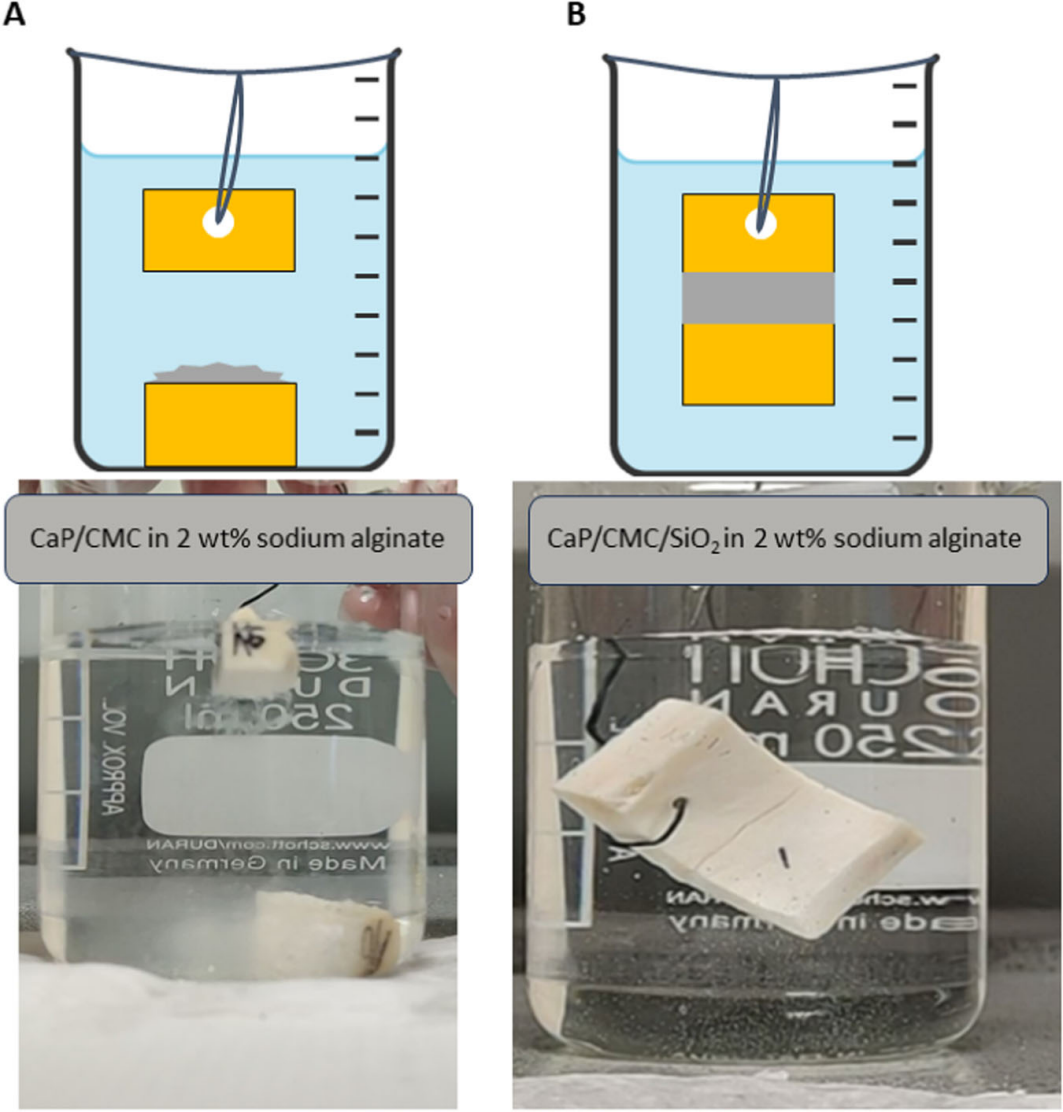
Schematic representation of different test setups to assess the adhesive strength after gluing previously fractured and then joined bone fragments. **A** CaP/CMC nanoparticles in a 2 wt% sodium alginate hydrogel gave a good adhesion in air but a poor adhesion after immersion, underscoring the importance of the silica shell. The glued bone fragment fell off immediately. **B** CaP/CMC/SiO_2_ nanoparticles in a 2 wt% sodium alginate hydrogel gave a good adhesion in air and also in water. Note the glued interface between the bone parts (diagonal from lower left to upper right)

In subsequent experiments, bone fragments were mechanically broken, resulting in a rough fracture interface which resembles the natural situation after a bone fracture. The nominal contact area was *A* = 1 to 3 cm^2^, depending on the bone sample used. The glued bone parts were transferred within 30 s into a water-filled beaker to subject them to the most challenging gluing condition, i.e., full immersion in water (Fig. 12). A water-based paste of CaP/CMC/SiO_2_ nanoparticles showed promising adhesive properties in the dry state (Fig. 11) before immersion in water but failed immediately as the paste came into contact with water. CaP/CMC nanoparticles without silica shell showed no adhesive strength in water, neither as aqueous dispersion nor as hydrogel within a 2 wt% sodium alginate solution. A sodium alginate solution without calcium phosphate nanoparticles also showed no significant adhesion in water. A 0.5 wt% sodium alginate solution together with CaP/CMC/SiO_2_ nanoparticles led to a short adhesion time below one minute. With increasing concentration of sodium alginate in the hydrogel, the adhesion time increased considerably. The hydrogel based on CaP/CMC/SiO_2_ nanoparticles in 2 wt% sodium alginate showed a long-term stability of more than three months under permanent immersion in water.

The results of the immersion experiments are summarized in Table 3.

**Table 3:** Performance of the hydrogel paste to glue fractured bone fragments (contact area 1 to 3 cm^2^) after complete immersion in water according to Fig. 12

Paste system	alginate only (2 wt%)	CaP/ CMC without alginate	CaP/ CMC/ SiO_2_ without alginate	CaP/ CMC with 2 wt% alginate	CaP/ CMC/ SiO_2_ with 0.5 wt% alginate	CaP/ CMC/ SiO_2_ with 1 wt% alginate	CaP/ CMC/ SiO_2_ with 2 wt% alginate
water in the paste (wt%)	98.0	84.4	84.6	49.2	See Table 1		
alginate in the paste (wt%)	2.0	—	—	1.0			
CaP/CMC/SiO_2_ in the paste (wt%)	—	—	15.4	—			
CaP/CMC in the paste (wt%)	—	15.5	—	49.8			
Time until failure	<1 min	0	0	<1 min	<1 min	≈15 min	>3 months

Only the formulations of alginate with CaP/CMC/SiO_2_ nanoparticles gave a sufficient stability after immersion. A long-term stability ( > 3 months) was achieved with the hydrogel containing 2 wt% alginate

**Fig. 13:**
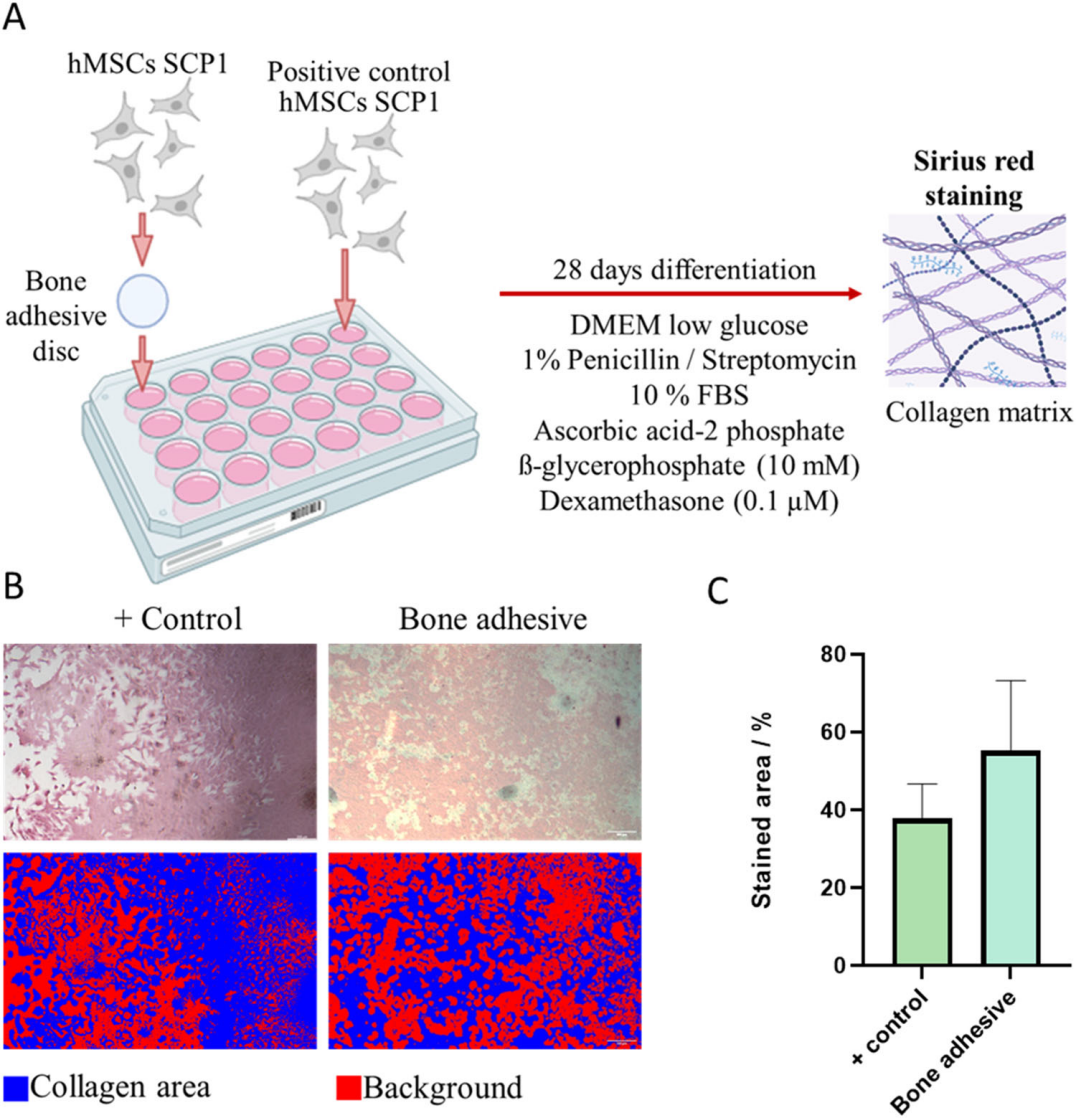
Experimental design for the evaluation of the effect of the bone adhesive paste on osteoblast differentiation. **A** Seeding hMSCs on a bone adhesive disc or directly in the cell culture well. Collagen inside the extracellular matrix was stained by Sirius red. **B** Images of Sirius Red staining on plastic (positive control) and on bone adhesive discs. **C** Quantification of collagen deposition revealed a positive differentiation of hMSCs on bone adhesive discs

Finally, it was tested whether the nanoparticle-loaded hydrogel was biocompatible in contact with osteoblasts (Fig. 13). Solid discs prepared from CaP/CMC/SiO_2_ nanoparticles were used as substrates for cell seeding. An unchanged differentiation of osteoblasts in the presence of the bone adhesive is important to ensure proper bone healing and remodelling [38]. Sirius red was used to stain collagen deposited by osteoblasts to confirm their differentiation. We found no significant difference in the collagen deposition as expressed by the percentage of the stained area on the bone adhesive surface compared to the control (cell culture well alone), i.e., 55 ± 16% and 38 ± 8%, respectively (unpaired *t* test: *p* = 0.059).

## Discussion

The particle-filled hydrogel can be considered as internally well-structured and highly associated according to the rheological experiments. It is rapidly crosslinking due to the release of calcium ions from calcium phosphate nanoparticles but remains liquid and viscous for application as adhesive. There is no indication for a particle separation from the gel during storage. The paste based on 2 wt% sodium alginate shows the highest degree of cross-linking as indicated by the highest moduli *G*’ and *G*” (Figs. 3 and 4) and was therefore chosen for subsequent gluing experiments. Clearly, there is a lower limit of the alginate concentration for a sufficient crosslinking of the hydrogel. The decrease in viscosity at increasing shear rate (Fig. 5) can be explained by disentanglement of the sodium alginate due to its high molecular weight of 300 to 350 kDa [35, 39]. During shearing, previously entangled and disordered macromolecules are arranged into the direction of shearing. This leads to a rearrangement of the disordered structure which reduces the flow resistance and leads to a lower viscosity. Furthermore, agglomerates are broken up into smaller particles, which has the same effect. For the envisioned application as bone adhesive this decrease of viscosity under application of shear stress is favourable as it facilitates the administration under shear stress with a syringe into the operation site. This corresponds to tests of the injectability from a syringe that showed no visible solid–liquid separation. The hydrogel is strongly thixotropic (Fig. 6), i.e., it reversibly liquefies under mechanical shear stress which is advantageous for an application with a syringe (Fig. 8).

The stabilization of the CaP/CMC nanoparticles with a silica shell and the presence of a highly concentrated sodium alginate solution are crucial for adhesive properties of the paste in the wet state. We assume that this is due to the inhibition of swelling of the hydrophilic CMC in water [40] that otherwise would lead to local swelling pressure and internal rupture of the glue layer. Although such an adhesive performs well in the moist state (Fig. 11), the adhesion immediately fails upon immersion in water (Fig. 12). CMC [40] (about 0.5 wt%) and calcium phosphate [41] (about 20 wt%) as major components of the paste are all biocompatible and biodegradable. The content of alginate [18] is about 1.6 wt% for the formulation based on a 2 wt% alginate solution and can be considered as well biodegradable, given the low concentration in the paste. Most of the particle-filled hydrogel is water (about 80 wt%; Fig. 7 and Table 1) which is clearly beneficial as it minimizes the presence of foreign material in the bone defect.

The cell-biological results with hMSCs (Fig. 13) indicate that the bone adhesive does not impair the osteoblast differentiation and the formation of extracellular matrix. This underscores the beneficial effect of calcium phosphate nanoparticles which are osteoconductive and perform the same action as calcium phosphates in bone substitution materials [31, 33].

The material must be sterilized before a clinical application. It may be possible to perform the whole synthesis under sterile conditions as it does not involve complex chemical operations. An alternative is gamma-sterilization which should not harm the components. In contrast, autoclaving would lead to hydrolysis of the nanoparticle components and the alginate gel and is therefore not possible.

The mechanical tests were carried out with cortical bone as this permits a better interpretation of the results. In that case, the interface is geometrically defined, flat, and mostly non-porous. In contrast, gluing cancellous bone would lead to the penetration of the paste into the pores. Besides the requirement for more paste, it would raise considerable questions whether the failure after mechanical loading was due to adhesive rupture at the bone – paste interface or to cohesive rupture inside the paste or the bone trabeculae. However, it can be safely assumed that the developed bone adhesive will be applicable for both cortical (solid) and cancellous (porous) bone, given the easy injectability during surgery with a syringe.

The adhesion strength of the paste is about 84 kPa in the moist state. This is lower than those reported for synthetic polymers (0.3 to 9 MPa [3]) and biobased adhesives (0.3 to 62 MPa [3]). It also does not reach the performance of phosphoserine-based injectable calcium phosphates (2 to 5 MPa [21, 22, 42]) and magnesium phosphates (6.6 to 7.3 MPa [23]). The adhesive strength of the nanoparticle-loaded alginate gel is clearly below what is needed for a loaded defect (although no threshold value has been defined so far [3]). However, the adhesion properties should be sufficient to join bone fragments under low mechanical stress. Therefore, it is envisioned that this bone adhesive can find application to join bone fragments in an operation site where osteosynthesis by mechanical devices is not possible, e.g., in a fracture in the hand or the foot. The thickness of the paste layer of about 0.2 mm corresponds to a volume of paste of 20 µL per mm^2^ glued area. This low amount of foreign material that is brought into such a defect (about 4 mg in 20 µL of paste for 1 mm^2^, most of it being calcium phosphate) leads a minimum irritation of the surrounding tissue. This, together with a high water content of 80% will facilitate the ingrowth of bone cells. It is also well conceivable to enhance bone growth by loading the nanoparticles with bioactive compounds like DNA for transfection of BMP or VEGF, siRNA against inflammatory chemokines or bisphosphonates as we have demonstrated earlier [43–47]. Of course, this would convert the material to a much more expensive and more regulated medical product or a drug [48].

## Conclusion

A particle-loaded hydrogel prepared from an aqueous solution of 2 wt% sodium alginate and CMC-stabilized silica-coated calcium phosphate nanoparticles can serve as bone adhesive with minimum content of foreign material. The paste was stable after mixing as long as it does not dry out and can be applied without any time constraints for bone fixation. The yield strength was about 84 kPa after gluing moist bone fragments. Upon complete immersion in water, the adhesion between previously fractured and then glued bone fragments was stable for at least three months. The presence of calcium phosphate is expected to enhance bone growth at the glued interface, indicated by osteoblast differentiation during in-vitro cell culture experiments. Although the adhesion strength is still insufficient to fix bone in a mechanically loaded fracture, it is well conceivable to apply the hydrogel to prevent micromovement after osteosynthesis or to stabilize fragments in a bone fracture site where osteosynthesis is not geometrically possible. Thus, it constitutes a biomaterial that can fill bone defects and is also able to keep bone fragments together, unlike a purely ceramic bone filler.
